# Role of Internet of Things (IoT), Artificial Intelligence and Machine Learning in Musculoskeletal Pain: A Scoping Review

**DOI:** 10.7759/cureus.37352

**Published:** 2023-04-09

**Authors:** Fatima Hasan, Abhay Mudey, Abhishek Joshi

**Affiliations:** 1 Community Medicine, Jawaharlal Nehru Medical College, Datta Meghe Institute of Medical Sciences, Wardha, IND

**Keywords:** exercise adherence, machine learning, internet of things (iot), artificial intelligence, musculoskeletal pain, pain

## Abstract

Artificial intelligence (AI), Internet of Things (IoT), and machine learning (ML) have considerably increased in numerous critical medical sectors and significantly impacted our daily lives. Digital health interventions support cost-effective, accessible, and preferred interventions that meet time and resource constraints for large patient populations. Musculoskeletal conditions significantly impact society, the economy, and people's life. Adults with chronic neck and back pain are frequently the victims, rendering them physically unable to move. They often experience discomfort, necessitating them to take over-the-counter medications or painkilling gels. Technologies driven by AI have been suggested as an alternative approach to improve adherence to exercise therapy, which in turn helps patients undertake exercises every day to relieve pain associated with the musculoskeletal system. Even though there are many computer-aided evaluations available for physiotherapy rehabilitation, current approaches to computer-aided performance and monitoring lack flexibility and robustness. A thorough literature search was conducted using key databases like PubMed and Google Scholar, as well as Medical Subject Headings (MeSH) terms and related keywords. This research aimed to determine if AI-operated digital health therapies that use cutting-edge IoT, brain imaging, and ML technologies are beneficial in lowering pain and enhancing functional impairment in patients with musculoskeletal diseases. The secondary goal was to ascertain whether solutions driven by machine learning or artificial intelligence can improve exercise compliance and be viewed as a lifestyle choice.

## Introduction and background

A wide range of conditions that impact bones, joints, and soft tissues are referred to as "musculoskeletal conditions". Over 100 different ailments, diseases, and syndromes make up musculoskeletal conditions, impacting people's quality of life, capacity to do daily tasks and activities of daily living (ADLs). Low back pain (LBP) and osteoarthritis (OA) are the most typical of these diseases. The most prevalent symptoms for the individual are pain, stiffness, knot formation, weariness, decreased movement, and loss of dexterity. Although the expense of treating musculoskeletal problems varies from country to country, it generally requires time and money. Musculoskeletal disorders are bothersome and typically hinder a person's quality of life [1]. An integrated, strategic approach that offers efficient and accessible forms of health service delivery on a community level is urgently needed to decrease the prevalence and growing impact of musculoskeletal impairment. [1] The delivery of health and exercise interventions via the Internet has the potential to be more affordable while also increasing patient acceptance and satisfaction. Patients encounter various obstacles to receiving in-person training, including travel expenses, time away from jobs or caregiving responsibilities, the cost of treatment if under or uninsured, and restricted access to healthcare providers in remote locations. Delivery systems based on the Internet can overcome these obstacles and pave the way to a new world where attaining positive health is not unachievable [2].

One way to achieve this goal is through wireless and mobile digital health solutions. Digital health interventions offer ways to address problems with the healthcare system and healthcare delivery care that is occasionally not available and has the potential to improve the sustainability and quality of musculoskeletal health services. The World Health Organization (WHO) has just released guidelines that classify digital health interventions as a tool to address various issues. The organization is also trying to standardize the terminology used by the many communities involved in digital health so that people can be aware of the available online resources. With this in mind, "digital health interventions" refers to patient interventions provided through apps, websites, or web-based software. Digital health interventions can provide accessible technology at home, which is more convenient, along with all the necessary patient education and self-management interventions available all the time for musculoskeletal populations, delivered via apps or web-based platforms. However, there are problems facing the implementation of digital health interventions. The major drawback is the failure to evaluate the situation appropriately before generalizing it. Additionally, there is a conflict between the rapid development of digital therapies and the gradual movement of more conventional clinical trial outcomes into clinical practice [1].

Artificial intelligence (AI), machine learning (ML), and Internet of Things (IoT) have been used in various medical specialities, including radiology, dermatology, cardiology, and mental health. Compared to other medical areas, the application of AI and ML in the orthopaedic field is still in its early stages [3]. Therefore, the primary goal of this research was to justify the increased use of digital interventions in patients with musculoskeletal issues. This article also outlines several effective web-based programmes that serve as exercise training guides and might be used as examples to follow through and bring about lifestyle changes.

## Review

Methodology

**Figure 1:**
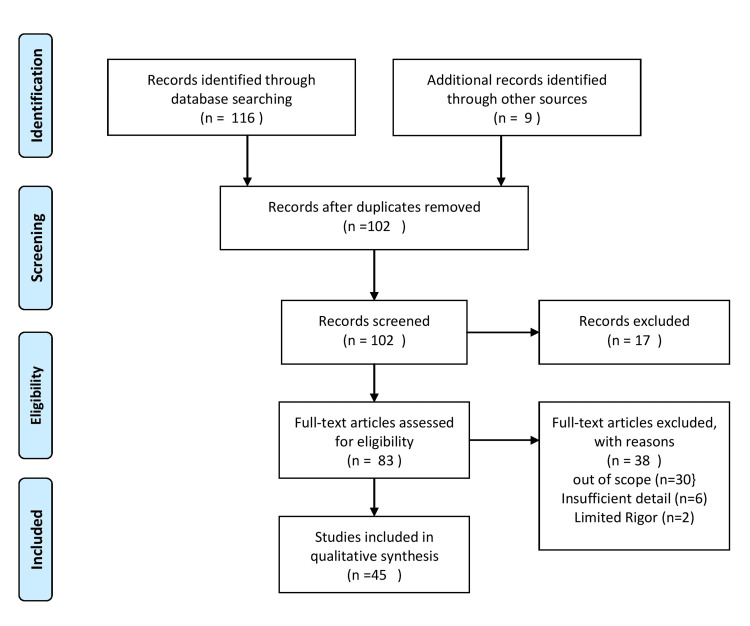
Inclusion and exclusion criteria of the study

An online search was conducted using PubMed and Google Scholar to find articles on using AI, ML and IoT to reduce musculoskeletal pain. Key Medical Subject Headings (MeSH) search terms such as "musculoskeletal pain", "artificial intelligence", "machine learning", "internet of things", and "exercise adherence" were used. They were all used interchangeably and in combination to obtain the desired results. Boolean operators "OR" and "AND" were used to combine search terms. Forty-five free articles were retrieved for review and had full-text availability. Articles ranging from 2010 to 2022 emphasized the increased usage of digital interventions to alleviate musculoskeletal pain. Figure 1 shows the inclusion and exclusion criteria.

This review's main goal was to ascertain whether digital health interventions positively impacted musculoskeletal pain and functional impairment in patients with musculoskeletal diseases. Self-management and rehabilitative interventions can be delivered through the Internet with success [2]. The findings indicate that there are specific recently created digital interventions using high-tech technologies that we can implement in our daily activities by adhering to a pattern that would aid individuals in finding relief from their suffering. Nine out of 19 studies reported substantial benefits, providing evidence for the usefulness of digital health interventions in reducing pain [1,4-14].

Employees with health issues such as discomfort, difficulty walking, and gait issues incur high medical care costs and low job productivity, characterized by absenteeism and presenteeism [15]. AI assists in keeping a digital record of the patients' issues and supports them through apps to enhance adherence to the exercises that patients typically avoid for various reasons, including a lack of time, an increased workload, and mood issues. The geoinformation system (GIS) aids in locating super-speciality centres and remote hospitals for those in need. It is crucial to stick to a specific intervention to relieve musculoskeletal pain, so some common treatments include exercise, medication, or surgery if the issue is severe. Musculoskeletal pain can be significantly reduced with the help of physiotherapists. Physiotherapy (PT) is underutilized partly because of problems with healthcare access, particularly for uninsured or underinsured patients and those who live in medically underserved areas. Lower socioeconomic class persons probably have less access to physical therapists or supervised exercise programmes. Therefore, they also have more cases of musculoskeletal pain [16]. The following technological breakthroughs are currently being used to diagnose and treat various medical issues.

Artificial intelligence

Through innovative care delivery strategies, well-informed decision-making, and the promotion of patient engagement, the mathematical engineering discipline of artificial intelligence has the potential to improve healthcare [17]. The many modalities of musculoskeletal imaging, such as dual-energy X-ray absorptiometry (DXA), radiography, computed tomography (CT), and magnetic resource imaging, have all been subjected to artificial intelligence algorithms (MRI). Applications for DXA include femoral segmentation for assessing bone density and the diagnosis of osteoporotic vertebral compression fractures [18]. AI helps recognize, track, and use its complicated format in situations that were previously difficult to notice. How an AI-integrated app assists those with chronic pain of any kind is shown in Table 1 [19].

**Table 1:** Functioning of an AI app AI, artificial intelligence Source: Reference [19]

Roles of an AI-integrated app	How?
1. Track symptoms as they occur, noting the initial symptoms	Patients find it convenient and sometimes more accurate to track their symptoms like pain in the joints, stiffness, buckling of their knees using mobile apps than they do using manual approaches. The doctors could ask the patients questions like how frequently they experience pain, which position alleviates it, etc. Patients and their doctors can better understand their pain and begin to recognize patterns by keeping a comprehensive record of their symptoms and daily activities.
2. Identify environmental factors that influence pain levels	AI helps monitor and track pain triggers, assisting patients and healthcare professionals to identify the most effective coping mechanisms. Using in-the-moment tracking, patients and their doctors may identify the environmental elements like workplace hazards, occupational stress, cold temperature, etc., that affect pain levels and take appropriate action.
3. Receive forewarning psychological symptoms of pain, such as depression, anxiety, and insomnia. Detecting how much water patients are consuming also helps, and also, whether they spend a lot of time not exercising	New AI-enabled tools can help therapists monitor patients' mental health and spot warning signs of more severe conditions. The tool captures a brief voice sample, and an algorithm analyzes the patient's voice for symptoms of depression or anxiety. This tool helps monitor patients daily by their concerned therapists or guardians, which allows them to assist individuals in need and generates more information for therapists to discuss with patients during sessions. A therapist accessing data from an AI app can comment, "It looks like you were struggling on Tuesday," "What do you suppose occurred?", even if the meeting is scheduled on a good day. The tool also helps in methodically examining patients' sleep patterns and keeping track of the daily workouts they perform and the things they avoid.
4. Walk patients/care seekers through alternative therapies	In cognitive-behavioural therapy, a patient and therapist collaborate to assist the patient in overcoming negative thought patterns and creating coping mechanisms.
5. Introduce patients to peers who can support and mentor them	Through peer-support apps, people with chronic pain can connect with other people. While therapists cannot always be reached, other people with chronic pain can. These kinds of technologies can close the essential gaps between appointments.

Brain imagining

Recent developments in structural and functional brain imaging have shown brain anomalies in disorders associated with chronic pain that can be used to categorize illnesses. Machine learning approaches have been used more frequently since analyzing complex and multivariate brain imaging data can be challenging. Machine learning aims to train specific classifiers to efficiently recognize variables of interest in brain MRIs [20]. MRI examines the biomarkers that aid in brain imaging, which in turn examines voluminous neuroimaging literature describing the crucial function of specific brain regions in the sensory, affective, and cognitive-evaluative domains (e.g., thalamus, primary and secondary somatosensory cortex posterior insula, basal ganglia, anterior insula, hypothalamus, anterior cingulate cortex, amygdala, hippocampus) [20].

Machine learning

Machine learning is a specific type of artificial intelligence that can be used to anticipate outcomes and automate decision-making based on patient data [17]. The best way to manage chronic pain is by figuring out the disease's underlying causes and enabling the personalization of treatments. ML does that by using facial apprehension, medical imagery, and brain signals to detect pain levels. Because ML is so good at seeing patterns, rules, and causal relationships in massive datasets, these are areas where it is anticipated to be helpful. Large and complicated datasets can be utilized to derive insights, generate predictions, and make judgements using ML algorithms, which learn from data without being explicitly programmed. These analyses can improve the diagnosis, prognosis, and treatment of diseases in healthcare. Large quantities of pertinent training data are required to produce reliable ML models. A greater use of information systems in healthcare generates enormous volumes of health data and makes ML research easier, which could lead to the realization of person-centred healthcare. In addition, as people use wearables and health applications more frequently for self-management, a lot of health-related data is being produced outside the traditional healthcare industry. ML algorithms may utilize this broad range of health data, made both in and out of clinical settings, to aid in studying chronic pain [21]. In this manner, ML aids in providing a tool for self-management and gathering data to highlight the things we overlook.

Internet of Things

New technological paradigms have emerged as a result of recent developments in sensing and processing technologies, such as the Internet of Things. To deliver specialized services for process automation and remote monitoring, it enables various devices with distinct identities to share information. IoT is made up of embedded sensing, transmission, and processing technologies that can be utilized for simultaneous autonomous, remote, and real-time monitoring. Electronic diaries are one example of how IoT-enabling technologies are being adopted for pain evaluation and treatment. With the development of wireless mobile devices like smartphones, healthcare professionals can easily upload patient pain data for analysis. Intriguingly, smartwatches don't draw as much attention to their usage for assessing and managing pain as smartphones and tablets have [22]. India's most widely utilized pain evaluation and management technologies include wireless sensor networks (WSNs), soft computing tools, and intelligent mobile devices [23,24]. Knowledge-based systems (KBSs) have recently been proposed as a potent tool for managing and assessing pain. Not only would they provide quantitative pain monitoring, but they might also help medical practitioners make decisions. In IoT, pain is automatically assessed and managed with a focus on several physiological data or facial expressions as pain indicators. Cloud computing is an ideal candidate for providing long-term storage of patient-related pain data and giving medical practitioners diagnostic information. IoT's limitations include security and privacy concerns. Healthcare professionals' limited understanding of IoT technologies and the need for knowledge about the advantages and hazards of using these tools are two potential barriers to adopting the IoT paradigm for pain evaluation and management [24].

Evidence-based web-based training programs for exercise

There are successful applications that offer web-based fitness schedules. They nudge people to keep up with exercise, keep track of their suffering, and support self-management to lessen the pain.

Internet-Based Exercise Training (IBET) Program

The primary problems influencing patients' quality of life who have knee OA are acknowledged to be chronic pain and reduced physical function [25]. Visual Health Information and a multidisciplinary team, which included physical therapists, doctors, and patients, created the IBET programme [16]. The IBET programme has the following features: (1) tailored exercises based on present activity, function, and discomfort measurements, as well as an algorithm that places individuals in one of seven different exercise levels; exercise programmes call for strengthening, stretching, and aerobic movement; (2) exercise progression advice based on repeated assessments of discomfort and function; (3) a video display of exercises (together with images) to show how to conduct exercises properly; (4) automated reminders to visit the website and stay active if users last logged in for seven days; (5) progress monitoring, including time-varying pain, function, and exercise graphs [16]. IBET should be utilized as a lifestyle tool to prevent knee-centric discomfort and as a technique to impose self-awareness and discipline on oneself.

Therapeutic Exercise Resource Center (TERC) for OA

The first complete web-based Therapeutic Exercise Resource Center for OA was created to assess, recommend, track, and modify therapeutic exercise regimens for people with knee OA. In this system, a tailored regimen, including strength, flexibility, and aerobic exercises, is produced based on the data given by the patient and used to target condition-specific neuromuscular deficiencies and enhance knee OA symptoms and general health. Healthcare professionals can use the TERC to encourage patients with knee OA to exercise [2]. According to the most recent standards of care and clinical practice recommendations, the exercises in the TERC include strength and flexibility exercises as well as suggestions for cardiovascular activities [26-30]. The quadriceps, hamstrings and gluteal muscles are primarily worked during strength and flexibility exercises, while a progressive walking programme is recommended for cardiovascular activity. To encourage proper technique, the TERC shows motion-captured animations of each strength and flexibility exercise and static photos of each customized training programme. The TERC uses the regulate-prescribe-monitor-adjust option to identify the issue and provides important recommendations to address it [2].

Web-Based Intervention to Promote Older Adults' Physical Activity: 'Active After 55'

The principle of planned behaviour provided the foundation for Active After 55, which asserts that perceptions of behavioural control (i.e., self-efficacy), social standards, and attitudes toward a behaviour cause behavioural intention and modification [31-33]. A substantial body of research evidence supports the importance of self-efficacy in adopting and maintaining health-promoting exercise activities for adults [34-36]. Recent research has demonstrated that this theory's interventions significantly affect behaviour in web-based interventions [37]. Therefore, those who are in need of assistance and are eager to experiment with and treat themselves using technology may find value in the web-based approach.

Internet Delivery of Animated Rehabilitation Exercise

The proposed project develops and assesses a web-based system that lets therapists send animated therapy exercises to a patient's home through the Internet. The patient can access the animated activities on their computer by clicking on a link the therapist sends them after selecting them from a menu. The objectives are to adjust the interface and manner in which the animations are delivered using the evaluation of the prototype and input from users and focus groups. The application will be integrated with software that prescribes exercises, and a website including more than 1200 animated rehabilitation exercises will be created as the final result [38].

Biofeedback

Utilizing a sensing platform, exercise biofeedback systems collect and analyze data to provide users with helpful information about their performance [39]. To determine a person's degree of stress, biofeedback measures their muscle tension using electromyography (EMG), hand temperature (blood flow), heart rate, breathing rate, and skin wetness (technically known as galvanic skin response or GSR). By combining relaxing techniques with the knowledge given, a person can take deliberate control over these measures. EMG biofeedback can also restore muscle function or teach people how to use their muscles more effectively. Treatment for peripheral biofeedback can frequently be finished in 10 sessions or fewer. According to a review by the Association for Applied Psychophysiology and Biofeedback, peripheral biofeedback helps treat the following chronic pain conditions: abdominal pain, complex regional pain syndrome/reflex sympathetic dystrophy (CRPS/RSD), fibromyalgia, jaw pain (temporomandibular disorder or TMD), low back pain, migraine and tension headaches, neck pain, and phantom limb pain [40,41].

Depending on one's pain and its likely cause, a biofeedback provider can test several functions using electrical sensors attached to the patient's body. Thanks to that information, chronic pain may be lessened in many ways. One's involuntary responses to stress and pain can be changed by practicing mindfulness and breathing exercises, which a biofeedback therapist can teach. An EMG session, for instance, can show that a particular muscle in the body is taut. That can imply that it contributes to the pain or is even the leading cause. The biofeedback therapist can teach methods to relax that muscle throughout several sessions; in addition to providing physical symptom alleviation, observing how the muscle untenses in response to these relaxation techniques can offer one a sense of positive agency over the pain that previously felt out of control [42]. The meta-analysis results show that biofeedback effectively treats persistent back pain and fibromyalgia [43,44].

Adherence to exercise therapy

Adherence is a crucial component that affects how the intervention procedure turns out. The success of the intervention process depends in large part on adherence. The effectiveness of the therapy decreases with decreasing exercise compliance. Hence, the patients must adhere to the protocol. The type of exercise does not have an impact on adherence. Still, other factors that contribute to adherence to exercise programs include their attractiveness, expert feedback and interaction, patient performance evaluation, and perception of expert support. Exercises are done under supervision, and review sessions and visual or auditory aids are also beneficial. Digital interventions are more likely to be used when additional support is provided, such as phone calls, email reminders, and text messages [15].

Use of chatbots to improve adherence

Currently, digital health applications for computers, smartphones, and tablets are generally accepted and reasonably priced, especially among young and middle-aged people [15]. To encourage people to continue doing their exercises, in Japan, an AI-assisted interactive health promotion system was developed; using a mobile messaging app Secaide, Ver. 0.9 (Travoss Inc., Tokyo, Japan), researchers tested people's adherence to exercise [15,45].

A randomized controlled trial revealed that the 12-week use of an AI-assisted health programme that offered one short-lived exercise routine per day significantly reduced the subjective symptoms of neck and shoulder pain/stiffness and low back pain when compared to baseline, with subjective improvement following the 12-week intervention in the intervention group compared to the control group. People's ability to access and use evidence-based health information for their healthcare is made possible by including mobile health and e-Health in health promotion activities. In other words, people can increase their health literacy. However, chatbots do not report dropout rates or language problems people might face that could hinder their routine. To overcome this, AI facilitates expert feedback requests from people. The effectiveness of digital interventions is increased when they are paired with human support. Apps with AI assistance that offer chatbots may reassure and support users. They assist with monitoring. Digital interventions encourage participants to keep working out and increase their adherence to the regimen, leading to a more significant reduction in symptoms. Deploying a chatbot as a healthcare support tool via a messaging app helps improve musculoskeletal symptoms in workers with neck/shoulder stiffness/pain [15].

Limitations

A few things cross our minds whenever we talk about smartphones and the increasing rise of technology. When looking for materials to seek out answers for people who are old and might face difficulties operating a smartphone, the unavailability of a specific solution was a significant setback. However, the difficulties could be overcome by providing them with links or making them practice how to use them. It should also be taken into consideration that the subjective responses by patients could have led to biases that might have impacted the results of some of the studies.

## Conclusions

This research highlights the potential for digital health interventions to reduce the adverse effects of musculoskeletal diseases on individuals, society, and the economy, which are only expected to worsen as our population ages. This driving force encourages the development of a system that should be practical, user-friendly, and solution-driven, and employing AI, IoT, and ML has been immensely helpful in this regard in the recent years. They assist in developing strategic plans to gradually lessen discomfort and also perform general health checks. They offer coping mechanisms, recommendations, peer support, accurate information, and feedback to all those in need. The potential of digital based health therapies has been explored in this research. The use of digital therapies has received a lot of favourable feedback, although some people may not be totally content with it. Despite this, the generalization of its usage still needs improvement and additional research to improve patient outcomes.

## References

[1] Hewitt S, Sephton R, Yeowell G (2020). The effectiveness of digital health interventions in the management of musculoskeletal conditions: systematic literature review. J Med Internet Res.

[2] Brooks MA, Beaulieu JE, Severson HH, Wille CM, Cooper D, Gau JM, Heiderscheit BC (2014). Web-based therapeutic exercise resource center as a treatment for knee osteoarthritis: a prospective cohort pilot study. BMC Musculoskelet Disord.

[3] Maffulli N, Rodriguez HC, Stone IW (2020). Artificial intelligence and machine learning in orthopedic surgery: a systematic review protocol. J Orthop Surg Res.

[4] Bennell KL, Nelligan R, Dobson F (2017). Effectiveness of an internet-delivered exercise and pain-coping skills training intervention for persons with chronic knee pain: a randomized trial. Ann Intern Med.

[5] Bennell KL, Nelligan RK, Rini C (2018). Effects of internet-based pain coping skills training before home exercise for individuals with hip osteoarthritis (HOPE trial): a randomised controlled trial. Pain.

[6] Bossen D, Veenhof C, Van Beek KE, Spreeuwenberg PM, Dekker J, De Bakker DH (2013). Effectiveness of a web-based physical activity intervention in patients with knee and/or hip osteoarthritis: randomized controlled trial. J Med Internet Res.

[7] del Pozo-Cruz B, Parraca JA, del Pozo-Cruz J, Adsuar JC, Hill J, Gusi N (2012). An occupational, internet-based intervention to prevent chronicity in subacute lower back pain: a randomised controlled trial. J Rehabil Med.

[8] del Pozo-Cruz B, Gusi N, del Pozo-Cruz J, Adsuar JC, Hernandez-Mocholí M, Parraca JA (2013). Clinical effects of a nine-month web-based intervention in subacute non-specific low back pain patients: a randomized controlled trial. Clin Rehabil.

[9] Del Pozo-Cruz B, Adsuar JC, Parraca J, Del Pozo-Cruz J, Moreno A, Gusi N (2012). A web-based intervention to improve and prevent low back pain among office workers: a randomized controlled trial. J Orthop Sports Phys Ther.

[10] Irvine AB, Russell H, Manocchia M (2015). Mobile-Web app to self-manage low back pain: randomized controlled trial. J Med Internet Res.

[11] Marangoni AH (2010). Effects of intermittent stretching exercises at work on musculoskeletal pain associated with the use of a personal computer and the influence of media on outcomes. Work.

[12] Mecklenburg G, Smittenaar P, Erhart-Hledik JC, Perez DA, Hunter S (2018). Effects of a 12-week digital care program for chronic knee pain on pain, mobility, and surgery risk: randomized controlled trial. J Med Internet Res.

[13] Shebib R, Bailey JF, Smittenaar P, Perez DA, Mecklenburg G, Hunter S (2019). Randomized controlled trial of a 12-week digital care program in improving low back pain. NPJ Digit Med.

[14] Toelle TR, Utpadel-Fischler DA, Haas KK, Priebe JA (2019). App-based multidisciplinary back pain treatment versus combined physiotherapy plus online education: a randomized controlled trial. NPJ Digit Med.

[15] Anan T, Kajiki S, Oka H, Fujii T, Kawamata K, Mori K, Matsudaira K (2021). Effects of an artificial intelligence-assisted health program on workers with neck/shoulder pain/stiffness and low back pain: randomized controlled trial. JMIR Mhealth Uhealth.

[16] Allen KD, Arbeeva L, Callahan LF (2018). Physical therapy vs internet-based exercise training for patients with knee osteoarthritis: results of a randomized controlled trial. Osteoarthritis Cartilage.

[17] Tack C (2019). Artificial intelligence and machine learning | applications in musculoskeletal physiotherapy. Musculoskelet Sci Pract.

[18] Burns JE, Yao J, Summers RM (2020). Artificial intelligence in musculoskeletal imaging: a paradigm shift. J Bone Miner Res.

[19] Asar A (2022). How AI and technology can help patients manage chronic pain. https://www.forbes.com/sites/forbestechcouncil/2020/09/25/how-ai-and-technology-can-help-patients-manage-chronic-pain/?sh=41294d854489.

[20] Boissoneault J, Sevel L, Letzen J, Robinson M, Staud R (2017). Biomarkers for musculoskeletal pain conditions: use of brain imaging and machine learning. Curr Rheumatol Rep.

[21] Jenssen MDK, Bakkevoll PA, Ngo PD (2021). Machine learning in chronic pain research: a scoping review. Appl Sci.

[22] Manini TM, Mendoza T, Battula M (2019). Perception of older adults toward smartwatch technology for assessing pain and related patient-reported outcomes: pilot study. JMIR Mhealth Uhealth.

[23] Rajesh M, Muthu JoanS, Suseela G (2012). A pain assessment and management app for a smart phone implementing sensors and soft computing tools. International Conference on Information Communication and Embedded Systems (ICICES).

[24] Prada EJA (2020). The Internet of Things (IoT) in pain assessment and management: an overview. Science Direct.

[25] Xie SH, Wang Q, Wang LQ, Wang L, Song KP, He CQ (2021). Effect of internet-based rehabilitation programs on improvement of pain and physical function in patients with knee osteoarthritis: systematic review and meta-analysis of randomized controlled trials. J Med Internet Res.

[26] Zhang W, Moskowitz RW, Nuki G (2008). OARSI recommendations for the management of hip and knee osteoarthritis, Part II: OARSI evidence-based, expert consensus guidelines. Osteoarthritis Cartilage.

[27] Fernandes L, Hagen KB, Bijlsma JW (2013). EULAR recommendations for the non-pharmacological core management of hip and knee osteoarthritis. Ann Rheum Dis.

[28] Richmond J, Hunter D, Irrgang J (2010). American Academy of Orthopaedic Surgeons clinical practice guideline on the treatment of osteoarthritis (OA) of the knee. J Bone Joint Surg Am.

[29] American College of Rheumatology Subcommittee on Osteoarthritis Guidelines (2000). Recommendations for the medical management of osteoarthritis of the hip and knee: 2000 update. Arthritis Rheum.

[30] American Geriatrics Society Panel on Exercise and Osteoarthritis (2001). Exercise prescription for older adults with osteoarthritis pain: consensus practice recommendations. A supplement to the AGS Clinical Practice Guidelines on the management of chronic pain in older adults. J Am Geriatr Soc.

[31] Ajzen I (1991). The theory of planned behavior. Organ Behav Hum Decis Process.

[32] Bandura A (1977). Self-efficacy: toward a unifying theory of behavioral change. Psychol Rev.

[33] Armitage CJ, Conner M (2001). Efficacy of the theory of planned behaviour: a meta-analytic review. Br J Soc Psychol.

[34] Irvine AB, Gelatt VA, Seeley JR, Macfarlane P, Gau JM (2013). Web-based intervention to promote physical activity by sedentary older adults: randomized controlled trial. J Med Internet Res.

[35] Blissmer B, McAuley E (2002). Testing the requirements of stages of physical activity among adults: the comparative effectiveness of stage-matched, mismatched, standard care, and control interventions. Ann Behav Med.

[36] Duncan TE, McAuley E (1993). Social support and efficacy cognitions in exercise adherence: a latent growth curve analysis. J Behav Med.

[37] Webb TL, Joseph J, Yardley L, Michie S (2010). Using the internet to promote health behavior change: a systematic review and meta-analysis of the impact of theoretical basis, use of behavior change techniques, and mode of delivery on efficacy. J Med Internet Res.

[38] (2022). Internet delivery of animated rehabilitation exercises. https://www.sbir.gov/sbirsearch/detail/319474.

[39] Argent R, Bevilacqua A, Keogh A, Daly A, Caulfield B (2021). The importance of real-world validation of machine learning systems in wearable exercise biofeedback platforms: a case study. Sensors (Basel).

[40] La Vaque TJ (2003). Neurofeedback, neuropathy and quantitative EEG. Handbook of Mind-Body Medicine for Primary Care.

[41] (2022). How to use biofeedback and neurofeedback for chronic pain. https://patient.practicalpainmanagement.com/biofeedback-neurofeedback-chronic-pain-treatment.

[42] (2022). Biofeedback therapy to help chronic pain?. https://www.everydayhealth.com/chronic-pain/biofeedback-therapy-can-help-chronic-pain/..

[43] Sielski R, Rief W, Glombiewski JA (2017). Efficacy of biofeedback in chronic back pain: a meta-analysis. Int J Behav Med.

[44] Glombiewski JA, Bernardy K, Häuser W (2013). Efficacy of EMG- and EEG-biofeedback in fibromyalgia syndrome: a meta-analysis and a systematic review of randomized controlled trials. Evid Based Complement Alternat Med.

[45] (2022). Stiff shoulders, back pain, SNS remote presenteeism countermeasures. https://www.secaide.me/.

